# Attitudes Toward Seeking Professional Psychological Help Among Health-Related and Non-Health-Related Students: A Cross-Sectional Study

**DOI:** 10.3390/healthcare14172704

**Published:** 2026-08-25

**Authors:** Nasser Ahmed Alkhamias, Salim Abdulraouf Almane, Mohammed Abdulaziz Albahli, Saud Mossab Alholiby AlbinZaid, Abdullah Duhailan Alduhailan, Abdullah Almaqhawi

**Affiliations:** 1 College of Medicine, King Faisal University, P.O. Box 400, Hofuf 31982, Al Ahsa, Saudi Arabia; 223021624@student.kfu.edu.sa (N.A.A.); 223026195@student.kfu.edu.sa (S.A.A.); 223036455@student.kfu.edu.sa (M.A.A.); 223041423@student.kfu.edu.sa (S.M.A.A.); 223036687@student.kfu.edu.sa (A.D.A.); 2 Department of Family and Community Medicine, College of Medicine, King Faisal University, Hofuf 31982, Al Ahsa, Saudi Arabia

**Keywords:** mental health, help-seeking, stigma, psychological distress, university students, Saudi Arabia

## Abstract

**Background:** Mental health disorders are an increasing concern in Saudi Arabia, including in the Eastern Province, where one study found that of the 42.5% of medical students who reported a clear need for mental health services, only 16.2% had actually used them. Stigma and other psychosocial factors are thought to shape help-seeking behavior, yet comparative studies remain scarce. This study compares attitudes toward seeking professional psychological help, and perceived stigma, between students in health-related and non-health-related programmes at King Faisal University. **Methods:** In this cross-sectional study, 589 students at King Faisal University, Saudi Arabia, were recruited through convenience sampling. Data were collected with an online, self-administered questionnaire covering sociodemographic characteristics and three validated Arabic instruments: the Attitudes Toward Seeking Professional Psychological Help–Short Form (ATSPPH-SF), the Stigma Scale for Receiving Psychological Help (SSRPH), and the Hopkins Symptom Checklist-25 (HSCL-25). **Results:** Of the 589 respondents, 580 (98.5%) were included in the analysis—279 (48.1%) health-related and 301 (51.9%) non-health-related students. Most were aged 18–21 (76.9%), Saudi nationals (96.9%), single (91.9%), and of middle income (79.2%). Attitudes toward seeking professional psychological help did not differ significantly between health-related and non-health-related students (Welch’s *t*(566) = −1.48, *p* = 0.138, Cohen’s d = −0.12, a negligible effect). Perceived stigma (SSRPH) was significantly higher among health-related students (10.85 vs. 10.16; *t*(578) = 2.56, *p* = 0.011). Perceived social stigma was inversely and significantly associated with attitudes toward seeking professional psychological help (r = −0.262, *p* < 0.001). Together, stigma and psychological distress (HSCL-25) explained a modest 7.9% of the variance in help-seeking attitudes (R^2^ = 0.079). **Conclusions:** Health-related and non-health-related students at this single university did not differ significantly in their attitudes toward seeking professional psychological help. Perceived stigma showed the stronger association with less favorable attitudes, whereas psychological distress was associated with a smaller, positive association. These findings, from a single-center convenience sample, point to a need for stigma-reduction efforts and confidential mental health services at King Faisal University.

## 1. Introduction

Mental health is a core part of overall well-being, shaping how people think, feel, and act. A World Health Organization systematic review and meta-analysis estimated the point prevalence of mental disorders—including depression, anxiety, post-traumatic stress disorder, bipolar disorder, and schizophrenia—at 22.1% specifically among populations living in conflict-affected settings [1]; this figure applies to conflict-affected populations and should not be read as a general global prevalence estimate. In general-population survey data, the lifetime prevalence of at least one of the 19 DSM-IV disorders assessed in one major survey was 34.2% [2]. Such problems, particularly anxiety and mood disorders, are highly prevalent among Saudi women [3]. University students are an especially vulnerable group, since they face academic pressure, social transitions, financial strain, and uncertainty about their future careers—stressors that can raise the risk of mental health problems during the university years [4].

A study of medical students in the Eastern Province illustrates the scale of the gap: 33.6% showed signs of depression, and although 42.5% expressed a clear need for mental health services, only 16.2% had used them in the previous 12 months [5]. This treatment gap is shaped by many factors, among them gender, living environment, academic performance, public stigma, and personal stigma [5]. Public stigma refers to the negative attitudes and beliefs the general population holds about mental illness, together with the discrimination experienced by those affected [6]. In Saudi Arabia, stigma surrounding mental illness remains one of the largest barriers to seeking professional psychological care, contributing to delayed treatment and underuse of the services that are available [7]. Personal stigma, by contrast, describes the prejudices and negative perceptions individuals hold about people with mental illness; as these biases spread, they can erode understanding, reduce access to care, and reinforce public stigma [6]. Two further constructs are commonly distinguished in this literature. Perceived stigma refers to an individual’s belief about how much society at large devalues or discriminates against people who seek or receive psychological help—that is, the person’s perception of others’ attitudes, irrespective of whether they personally endorse those attitudes. Self-stigma refers to the internalisation of such attitudes, whereby the individual applies the devaluing beliefs to themselves and experiences a resulting loss of self-esteem or self-worth for having sought help. These four constructs are related but conceptually distinct: public stigma resides in the population, personal stigma in the individual’s own endorsed beliefs about others, perceived stigma in the individual’s reading of the social environment, and self-stigma in the individual’s beliefs about themselves. The Stigma Scale for Receiving Psychological Help (SSRPH) used in the present study measures perceived stigma specifically—the respondent’s perception of how society views a person who receives psychological treatment—and does not assess public, personal, or self-stigma. Accordingly, the term “perceived stigma” is used throughout this manuscript when referring to the SSRPH outcome.

These four constructs are usually organised into a conceptual pathway rather than treated as independent influences. Corrigan and Watson’s widely cited account describes a progression in which an individual first becomes aware that society devalues people with mental illness (perceived stigma), may then come to agree with those beliefs (personal stigma), and may finally apply them to the self, with consequent loss of self-esteem and self-efficacy (self-stigma) [8]. Corrigan, Druss, and Perlick extended this model specifically to care-seeking, distinguishing the prejudice directed at people with mental illness from the stigma attached to the act of receiving treatment, and arguing that the latter operates through label avoidance—declining care in order to avoid being categorised [9]. Vogel and colleagues formalised the endpoint of this pathway by developing a dedicated measure of the self-stigma of help-seeking and showed that perceived public stigma is associated with help-seeking outcomes largely indirectly, through its internalisation as self-stigma and its expression in attitudes toward treatment [10]. Within this framework, attitudes occupy an intermediate position: they are shaped upstream by psychological distress, which creates the perceived need for care, and by the stigma the individual perceives in the surrounding social environment, and they in turn precede any intention or decision to consult a professional. The present study measures two of these upstream constructs (distress via the HSCL-25 and perceived stigma via the SSRPH) together with the attitudinal outcome (ATSPPH-SF); it does not measure personal stigma, self-stigma, intention, or actual service use, and the analyses reported below are therefore confined to the associations among distress, perceived stigma, and attitudes.

The empirical support for a stigma–help-seeking link is substantial but more nuanced than is often assumed. Clement and colleagues’ systematic review of quantitative and qualitative studies found stigma to be among the most frequently reported barriers to seeking help, with treatment stigma and internalised shame featuring especially prominently in accounts from students and young adults [11]. A subsequent meta-analysis by Schnyder and colleagues disaggregated the effect by stigma type and reported that personal stigma and help-seeking attitudes were associated with active help-seeking, whereas perceived public stigma showed no comparable association [12], a finding that converges with Eisenberg and colleagues’ earlier evidence from college students [13] and that bears directly on the interpretation of studies such as ours, which measure perceived rather than personal or self-stigma. Distress itself is a further and partly independent influence: in the original validation of the SSRPH, higher psychological distress was associated with less favourable rather than more favourable help-seeking attitudes [14], indicating that need and willingness do not move together in a simple fashion. Among health-professional students specifically, the case for attention is strong on prevalence grounds alone: Rotenstein and colleagues’ meta-analysis of 195 studies across 47 countries estimated a pooled prevalence of depression or depressive symptoms of 27.2% among medical students and found that only about one in six of those screening positive sought psychiatric treatment [15]. That combination of elevated need and low uptake, observed internationally, is what motivates the present comparison in a Saudi setting.

Attitudes and behaviors around mental health care vary by region and can be slow to change. Yet little research in Saudi Arabia compares the attitudes and perceived stigma of health-related and non-health-related university students regarding the use of mental health services. King Faisal University (KFU), in Al-Ahsa, offers a useful setting for examining these differences, given a diverse student body that spans a wide range of ages, academic fields, and socioeconomic backgrounds. This study therefore had three objectives: (1) to compare attitudes toward seeking professional psychological help, perceived stigma, and psychological distress between health-related and non-health-related students at KFU; (2) to examine the correlation between perceived stigma (SSRPH) and attitudes toward seeking professional psychological help (ATSPPH-SF); and (3) to identify independent correlates of help-seeking attitudes using multivariable linear regression, adjusting for sociodemographic and academic characteristics.

## 2. Materials and Methods

### 2.1. Study Design and Setting

This cross-sectional study was carried out between October 2025 and March 2026 among students at King Faisal University, Saudi Arabia, with the goal of assessing attitudes toward seeking professional psychological help among students in health-related and non-health-related programmes.

### 2.2. Study Population

The target population comprised all undergraduate students enrolled across the various academic years and majors during the 2025–2026 academic year at King Faisal University. Students who submitted incomplete questionnaires or declined to take part were excluded. Faculty members, administrative staff, and other institutional employees were also excluded so that the study focused exclusively on students. The primary, pre-specified group comparison classified respondents by broad academic track: the “health-related” group comprised the College of Medicine, College of Veterinary Medicine, College of Applied Medical Sciences, College of Dentistry, and College of Clinical Pharmacy (n = 279); the “non-health-related” group comprised the Applied College (foundation-year programs), Engineering, Computer Science and Information Technology, Law, Arts, Science, Sharia and Islamic Studies, Agricultural and Food Sciences, Education, and Business Administration (n = 301). A secondary, exploratory comparison contrasted the College of Medicine alone (n = 147) with all other colleges combined (n = 433); this comparison was not part of the original pre-specified hypothesis and is reported as exploratory throughout. We also note that 8 of the 580 analyzed respondents (1.4%) answered “no” to the screening item asking whether they were a King Faisal University student; they were retained in the primary analysis, and a sensitivity analysis excluding them (n = 572) is reported in the Results and did not change the substantive conclusions.

### 2.3. Sample Size and Sampling Technique

The required sample size was calculated using the single-population-proportion formula, which indicated that a minimum of 377 participants was needed. The parameters used in that original calculation were an assumed proportion of *p* = 0.50 (the most conservative value, maximising the required sample in the absence of a reliable local estimate for the outcome), an absolute precision (margin of error) of d = 0.05, a two-sided confidence level of 95% (Z = 1.96), and a design effect of 1.0 (simple random sampling assumed), giving an unadjusted requirement of 384 participants; applying a finite-population correction for the approximate size of the enrolled undergraduate population at King Faisal University yielded the reported figure of 377. In the end, 589 students took part, of whom 580 were analysed. We acknowledge that this calculation was framed around estimating a single proportion and was therefore not aligned with the principal inferential analyses of this study, namely the between-group mean comparison and the multivariable regression. To address this directly, we conducted a prospective (a priori-style) sensitivity power analysis based on the achieved group sizes and the number of model parameters, rather than on the observed effect sizes. For the primary between-group comparison of ATSPPH-SF scores (n = 279 vs. n = 301), a two-sided independent-samples *t*-test at α = 0.05 has 80% power to detect a standardised mean difference of d = 0.23 and 90% power to detect d = 0.27; the study was therefore adequately powered to detect small-to-moderate group differences, but not to detect differences smaller than approximately a quarter of a standard deviation. For the multivariable regression (N = 580), the design has 80% power at α = 0.05 to detect an effect size of f^2^ = 0.017 for a two-predictor model and f^2^ = 0.025 for a seven-predictor adjusted model, corresponding to increments in R^2^ of roughly 1.6% and 2.4%, respectively. Accordingly, the achieved sample was adequate for the regression analyses and for detecting small-to-moderate between-group differences, but a non-significant group comparison in this study cannot be interpreted as evidence of equivalence, since differences smaller than d ≈ 0.23 would likely have gone undetected. No power calculation reported here is derived retrospectively from the observed effect size.

Participants were recruited by convenience sampling. The online survey link was shared through WhatsApp groups across different colleges within the university, distributed by student representatives within each college’s official group. Because the invitation was circulated through group chats rather than sent to an enumerated list of individuals, the number of students who received or viewed the invitation is unknown and a response rate cannot be calculated. Multiple submissions from the same respondent were limited by the survey platform’s one-response restriction, but participation was not restricted to verified institutional accounts. This approach allowed data to be collected quickly, but the resulting sample should not be assumed to represent a broad or unbiased cross-section of the student body.

### 2.4. Study Variables

The primary outcome was attitudes toward seeking professional psychological help. Secondary outcomes included perceived stigma and symptoms of anxiety and depression. The independent variables were sociodemographic characteristics: age, gender, academic major, living area, and socioeconomic status.

### 2.5. Data Collection Instrument and Procedure

Data were collected using a structured, self-administered questionnaire in Arabic. The instrument was adapted from previously validated studies [7], with minor modifications to improve clarity and comprehensibility (Supplementary Table S1). It had two main sections:

- Sociodemographic characteristics: Age, gender, academic major, academic year, living area, and socioeconomic status (SES).
- Validated psychological scales:
- Attitudes Toward Seeking Professional Psychological Help–Short Form (ATSPPH-SF-Arabic) [16,17]: Attitudes toward professional help-seeking. The 10-item short form was derived by Fischer and Farina from the original 29-item scale [16]; its psychometric properties, including the debate over its dimensionality, have been examined in detail by Elhai and colleagues [17].
- Stigma Scale for Receiving Psychological Help (SSRPH-Arabic) [14]: Perceived stigma related to seeking psychological services, as originally developed by Komiya and colleagues [14].
- Hopkins Symptom Checklist-25 (HSCL-25-Arabic): Symptoms of anxiety and depression.

### 2.6. Pilot Study and Instrument Reliability

A pilot study was conducted before the main data collection to evaluate the clarity, comprehensibility, and reliability of the questionnaire. In the pilot, the ATSPPH showed moderate reliability (Cronbach’s α = 0.581) and the SSRPH showed acceptable reliability (Cronbach’s α = 0.692), while the HSCL-25 demonstrated excellent internal consistency (Cronbach’s α = 0.899). Items H3 and H15 of the HSCL-25 correlated negatively with the total scale score. Based on these findings, minor changes were made to improve the clarity and comprehension of the questionnaire.

All instruments had been validated in Arabic-speaking populations. Internal consistency was assessed using Cronbach’s alpha in Jamovi for the ATSPPH, the SSRPH, and the HSCL-25, with a value of ≥0.70 taken as acceptable. In the full sample, the ATSPPH showed moderate reliability (Cronbach’s α = 0.671; McDonald’s omega = 0.645); item-rest analysis of the correctly reverse-scored items showed a weak positive item-rest correlation for item 8 (r = 0.08) and item 4 (r = 0.13); an earlier calculation that entered item 8 in its raw, unreversed form yielded r = −0.08, which is the source of the negative value reported in an earlier draft of this manuscript—the total scores, group comparisons, and regression results reported throughout this paper already used the correctly reverse-scored item and are unaffected. An exploratory factor analysis of the ATSPPH-SF yielded three eigenvalues greater than 1 (2.83, 1.88, 1.09), with items 4 and 8 loading weakly (<0.06) on the dominant factor; a sensitivity analysis excluding item 8 (α = 0.696, ω = 0.683) is reported in Section 3.1 and did not change the substantive conclusions. All items were nonetheless retained in the primary analysis to preserve the scale’s original structure. The exploratory factor analysis was conducted on the 10 correctly reverse-scored ATSPPH-SF items in the full analytic sample (n = 580) using principal-axis factoring with varimax rotation. Sampling adequacy was satisfactory (Kaiser–Meyer–Olkin measure = 0.76) and Bartlett’s test of sphericity was significant (χ^2^ = 1143.4, df = 45, *p* < 0.001), confirming the correlation matrix was suitable for factor analysis. Applying the Kaiser criterion (eigenvalue > 1) retained three factors, accounting for 28.3%, 18.8%, and 10.9% of the total variance (58.0% cumulative for the initial eigenvalues; 22.0%, 12.7%, and 5.2%, 39.9% cumulative, for the rotated common-factor solution). Because the Kaiser criterion is known to over-extract, we additionally ran a parallel analysis against 500 random datasets of equivalent dimensions, which retained two factors rather than three. On the rotated solution, the first factor was defined by the positively worded help-seeking items (items 1, 3, 5, 6, and 7; loadings 0.60–0.73 in absolute value) and the second by the negatively worded self-reliance items (items 2, 4, 9, and 10; loadings 0.39–0.74 in absolute value), indicating that this structure largely reproduces the direction of item wording rather than distinct substantive dimensions. Items 4 and 8 loaded weakly on the dominant factor (0.06 and 0.10, respectively) and had the lowest communalities of the set. The complete rotated loading matrix, communalities, and item-rest correlations for all 10 items are provided in Supplementary Table S1. We note explicitly that the emergence of three eigenvalues greater than 1, the weak loadings of items 4 and 8 on the dominant factor, and the modest internal-consistency coefficients (α = 0.671, ω = 0.645) together indicate that the ATSPPH-SF did not behave as a strictly unidimensional scale in this sample. Summing all 10 items into a single total score therefore rests on the scale’s established scoring convention and on comparability with prior literature rather than on demonstrated unidimensionality in these data, and total-score findings should be interpreted with this caveat in mind. Because the Kaiser criterion is known to over-extract factors, we recommend that future work in this population evaluate the factor structure using parallel analysis and confirmatory factor analysis in an independent sample before the total score is treated as a unidimensional measure. The SSRPH showed acceptable reliability (Cronbach’s α = 0.776), and the HSCL-25 again demonstrated excellent internal consistency (Cronbach’s α = 0.934; no items showed a negative item-rest correlation in the full sample).

### 2.7. Data Management and Statistical Analysis

Data were managed in Jamovi (version 2.7.29) and Python (version 3.14.2, pandas, SciPy, statsmodels). Descriptive statistics summarized the sociodemographic characteristics and study variables: categorical variables were reported as frequencies and percentages, and continuous variables as means and standard deviations. Inferential analyses examined associations between the independent variables and attitudes toward seeking professional psychological help. Pearson’s correlation coefficient was used to examine the association between perceived stigma (SSRPH) and attitudes toward seeking professional psychological help (ATSPPH-SF) and independent-samples *t*-tests (Student’s and Welch’s, with Levene’s test guiding which is reported as primary) for continuous variables. Multiple linear regression was then used to identify independent correlates of attitudes toward help-seeking. An adjusted model additionally controlled for gender, age, academic year, socioeconomic status, nationality, and living area. Academic year was included as a covariate because the health-related and non-health-related groups differed markedly in their academic-year distribution (Table 1), making it a potential confounder of the group comparison; it was entered as a categorical variable with seven levels (preparation year, years 1–5, and internship year), with the preparation year as the reference category. Both R^2^ and adjusted R^2^ are reported for all regression models. Regarding missing data: the online questionnaire required a response to every item before submission, so no item-level missingness occurred among submitted questionnaires; participant-level exclusions comprised the 9 respondents (1.5% of 589) who declined consent, at which point the survey terminated before any further data were collected. All 580 retained participants therefore had complete data on every variable, and all models were fitted on the full analytic sample (N = 580) using complete-case analysis, with no imputation required. We verified this directly in the raw dataset: among the 580 retained respondents there were no missing values on any sociodemographic item or on any of the 40 scale items, so all models are based on the full N = 580. Residual diagnostics (Jarque–Bera normality test, Breusch–Pagan heteroscedasticity test, variance inflation factors, and Cook’s distance) were examined for all regression models, and heteroscedasticity-robust (HC3) standard errors were computed as a check. Sensitivity analyses re-ran the primary comparison and regression excluding ATSPPH-SF item 8 and excluding respondents who indicated they were not King Faisal University students; an exploratory interaction model tested whether student group moderated the associations of stigma and distress with attitudes. Statistical significance was set at *p* < 0.05.

### 2.8. Ethical Considerations

Ethical approval was obtained from the Deanship of Scientific Research at King Faisal University (Reference No. KFU-2025-ETHICS3722, consistent with the Institutional Review Board Statement below). Participation was voluntary, and informed consent was obtained electronically from all participants before data collection. Anonymity and confidentiality were maintained for all respondents throughout the study.

## 3. Results

Of the 589 respondents, 580 (98.5%) were retained for analysis the 9 excluded respondents (1.5%) had explicitly declined consent to participate, at which point the survey terminated with no further data collected from them. The retained sample comprised 279 health-related students (48.1%) and 301 non-health-related students (51.9%), all from King Faisal University (Table 2). Most participants were aged 18–21 (76.9%). The distribution across academic years differed by group: health-related students were most often in year 3 (37.6%), whereas non-health-related students were most often in year 1 (37.5%). The majority were Saudi nationals (96.9%), with 3.1% from other countries, and most lived in cities (75.2%) rather than villages (24.8%).

Across the whole sample, few students were employed (4.3%), while 95.7% were not; most were single (91.9%), with smaller proportions married (7.4%) or divorced (0.7%). By income, most reported middle income (79.2%), followed by low income (9.3%), high income (8.2%), and an “other” category (3.5%). As shown in Table 1, the two groups differed significantly in gender, age, academic year, and marital status (all *p* < 0.001), but not in nationality, living area, employment, or economic status (all *p* > 0.05); the gender and academic-year imbalances in particular are the reason these variables were included as covariates in the adjusted analyses reported in Section 3.4.

### 3.1. Help-Seeking Attitudes: Medical vs. Non-Medical Students

Levene’s test was significant for the ATSPPH-SF comparison (*p* = 0.039), indicating unequal variances; Welch’s *t*-test is therefore reported as the primary result: Welch’s *t*(566) = −1.48, *p* = 0.138, Cohen’s d = −0.12 (Student’s *t*(578) = −1.49, *p* = 0.137, reported for completeness). In short, no statistically significant difference in self-reported attitudes toward seeking professional psychological help was detected between health-related and non-health-related students (Table 3). Because no equivalence or non-inferiority testing was performed, this result should not be read as demonstrating that the groups are equivalent. The between-group difference in perceived stigma (SSRPH), by contrast, was statistically significant: health-related students scored higher (M = 10.85, SD = 3.22) than non-health-related students (M = 10.16, SD = 3.34), *t*(578) = 2.56, *p* = 0.011, 95% CI for the mean difference [0.16, 1.22], Cohen’s d = 0.21, a small effect.

### 3.2. Association Between Stigma and Help-Seeking Attitudes

Perceived social stigma (SSRPH total) was inversely correlated with attitudes toward seeking professional psychological help (ATSPPH-SF total): Pearson r = −0.262, *p* < 0.001 (df = 578). Greater perceived stigma was associated with less favorable help-seeking attitudes (Table 3).

### 3.3. Predictors of Help-Seeking Attitudes

A multiple linear regression predicting ATSPPH-SF total (N = 580) from SSRPH total and HSCL-25 mean explained 7.9% of the variance (R = 0.282, R^2^ = 0.079, adjusted R^2^ = 0.076) (Table 4).

Higher perceived stigma was independently associated with less favorable help-seeking attitudes: each one-point increase in SSRPH corresponded to a 0.43-point decrease in ATSPPH-SF total (β = −0.283, *p* < 0.001). Greater psychological distress was associated with slightly more favorable attitudes toward help-seeking (B = 0.88 per one-point increase in HSCL-25 mean; β = 0.104, *p* = 0.011).

Regression diagnostics for this model were satisfactory: variance inflation factors were 1.04 for both predictors (no multicollinearity), residuals approximated normality (Jarque–Bera *p* = 0.118) and homoscedasticity (Breusch–Pagan *p* = 0.584), and no observation exceeded conventional influence thresholds (maximum Cook’s distance = 0.037). Heteroscedasticity-robust (HC3) standard errors produced the same conclusions (stigma *p* < 0.001; distress *p* = 0.017).

### 3.4. Adjusted and Sensitivity Analyses

Because the health-related and non-health-related groups differed in gender and academic-year composition (Table 1), we re-estimated the group comparison in a multivariable model additionally adjusting for gender, age, academic year, socioeconomic status, nationality, and living area. Academic year was included in this adjusted model because the two groups differed pronouncedly in their academic-year distribution, making it a plausible confounder of the group comparison; academic year was entered as a categorical variable with the preparation year as the reference category. The adjusted association between student group and ATSPPH-SF total remained non-significant (B = −0.23, 95% CI [−1.11, 0.65], *p* = 0.609); adding perceived stigma and distress to this model did not change this conclusion (B = −0.14, 95% CI [−1.00, 0.72], *p* = 0.749). In the demographic model, male gender was independently associated with less favourable attitudes (B = −2.16, 95% CI [−3.07, −1.24], *p* < 0.001), and this association persisted after stigma and distress were added (B = −1.53, 95% CI [−2.45, −0.60], *p* = 0.001). No level of academic year differed significantly from the preparation year (all *p* ≥ 0.094), and socioeconomic status, nationality, and living area were likewise non-significant. Notably, once academic year was included, the association between age and attitudes reported in the previous version of this manuscript was no longer statistically significant (B = 0.24, 95% CI [−0.11, 0.59], *p* = 0.174), consistent with age and academic year carrying overlapping information; we therefore no longer report an independent age effect. The full adjusted model is presented in Table 5.

A post hoc variable not analyzed in the original study—prior personal use of psychiatric or psychological services (reported by 65 of 580 respondents, 11.2%)—was a strong independent correlate of more favourable attitudes (B = 3.25, 95% CI [2.01, 4.50], *p* < 0.001); once this variable was added to the fully adjusted model, the association between distress and attitudes was no longer significant (*p* = 0.383), suggesting the simpler model’s distress effect may partly reflect a greater history of help-seeking among more distressed students rather than a direct effect of current distress. The group comparison remained non-significant in this fuller model (B = −0.08, 95% CI [−0.92, 0.77], *p* = 0.859), and this model explained 16.7% of the variance (adjusted R^2^ = 0.142).

A sensitivity analysis excluding ATSPPH-SF item 8 reproduced the same pattern: the group comparison remained non-significant (Welch’s *t* = −1.03, *p* = 0.306, d = −0.09), and stigma and distress remained significant correlates of the reduced total score (R^2^ = 0.071, adjusted R^2^ = 0.068; Cronbach’s α for the 9-item version = 0.696). A further sensitivity analysis excluding the 8 respondents who indicated they were not King Faisal University students (n = 572) likewise left the group comparison non-significant (Welch’s *t* = −1.66, *p* = 0.097). Finally, an exploratory model testing whether student group moderated the stigma–attitude and distress–attitude associations found no significant interactions (group × stigma, *p* = 0.168; group × distress, *p* = 0.191), providing no evidence that these associations differ between health-related and non-health-related students.

The secondary, exploratory comparison between the College of Medicine (n = 147) and all other colleges combined (n = 433) was likewise non-significant, both unadjusted (*t* = −0.96, *p* = 0.340) and adjusted for demographics (including academic year), stigma, and distress (B = 0.53, 95% CI [−0.51, 1.57], *p* = 0.314).

## 4. Discussion

### 4.1. Help-Seeking Attitudes

Students in health-related programmes in this sample did not report more favorable attitudes toward seeking professional psychological help than their non-health-related peers. This comparison was not statistically significant (Welch’s *p* = 0.138, d = −0.12), with mean ATSPPH-SF scores of 24.70 for health-related students and 25.30 for non-health-related students. This absence of a detected difference does not establish that medical training has no effect on attitudes; it indicates only that this cross-sectional comparison did not detect one, and adjusting for gender, age, academic year, socioeconomic status, nationality, and living area did not change this conclusion (Table 5). With an overall mean close to the scale’s midpoint [16], the prevailing attitude across the student body was essentially neutral. This contrasts with a Korean study in which mental health literacy had a direct effect on help-seeking attitudes—an effect fully mediated by stigma [18]. Mental health literacy was not measured in the present study, so we cannot attribute the difference between samples to literacy; we note the comparison descriptively rather than as an explanation. Our results align closely with recent studies in Saudi Arabia, which likewise found uniformly low help-seeking attitudes [4,7,19,20]. Eisenberg et al. similarly reported that personal stigma was a significant negative predictor of help-seeking, whereas perceived public stigma was not [13].

These patterns admit more than one interpretation. Read alongside our regression findings, perceived social stigma was significantly and negatively associated with attitudes (β = −0.283, *p* < 0.001), though the cross-sectional design does not allow us to conclude that stigma actively suppresses attitudes. Notably, even when students from the College of Medicine—future physicians—were compared with all other majors, no significant difference in help-seeking attitudes emerged, consistent with the adjusted and exploratory-interaction analyses in Section 3.4.

### 4.2. Stigma

In the broad grouping, health-related majors (which include medicine, dentistry, pharmacy, applied medical sciences, and veterinary medicine, among others) reported slightly higher stigma than non-health-related majors (10.85 vs. 10.16). When the College of Medicine alone was compared with all other majors, the gap widened (11.31 vs. 10.21). AlSamhori et al. found that 84.7% of students held negative attitudes toward help-seeking, and that those who saw mental illness as embarrassing scored higher on stigma [21]. Although we did not find studies directly comparing stigma between health-related and non-health-related students, works such as those of Alluhaibi and Awadalla indicate that stigma is generally high in the Saudi sociocultural context [7].

In the regression, the SSRPH stigma score was a significant negative predictor (β = −0.283, *p* < 0.001), the stronger of the two correlates examined in a model that explained only 7.9% of the variance; many other potential barriers were not measured, so this should not be read as establishing stigma as the strongest barrier among all possible barriers—a finding consistent with several other studies [4,5,7,19,20,22]. Each one-unit increase in SSRPH, which measures perceived stigma specifically related to receiving psychological help (as distinct from public, personal, or self-stigma more broadly), was associated with a 0.43-point drop in help-seeking. These results are consistent with perceived stigma being an important correlate of less favorable attitudes among King Faisal University students, though the constructs of family shame or public stigmatization specifically were not measured here.

### 4.3. Psychological Distress

Regardless of how groups were defined, self-reported psychological distress (HSCL-25) was similar across the groups examined. In the broad grouping, the health-related track (M = 2.08) and non-health-related track (M = 2.06) were close (*p* = 0.657), as were the College of Medicine cohort (M = 2.04) and all other colleges combined (M = 2.08) in the isolated grouping. No statistically significant difference in self-reported distress was detected between groups. Because this is a cross-sectional observational comparison, these data cannot determine whether the medical curriculum does or does not contribute to anxiety and depressive symptoms, nor can they rank the relative contribution of academic major against other influences such as general academic transitions or developmental pressures; such influences were not measured here. The overall level of distress is consistent with other reports; Al-Johani et al., for example, found that 33.4% of participants showed high distress [23].

In this sample, no statistically significant between-group difference in self-reported distress was detected, whereas perceived stigma was significantly higher in the health-related group. These are two separate cross-sectional associations; the present design cannot establish that medical training causes, or fails to cause, either pattern. Our findings echo Alluhaibi and Awadalla, who reported that psychological distress was a significant positive predictor of help-seeking attitudes, with greater distress linked to more positive attitudes [7]. One explanation is that medical students internalize an image of the clinician as a strong, resilient caregiver, so that admitting psychological distress may feel like conceding professional incompetence or weakness.

In our data, the HSCL-25 distress score was a small positive predictor (β = 0.104, *p* = 0.011). This cross-sectional association does not establish that students delay help-seeking until distress outweighs stigma-related fear; the present data cannot speak to timing or sequence of care-seeking. Notably, once prior use of psychiatric or psychological services was added to the model, this association with distress was no longer significant, suggesting it may partly reflect a greater history of help-seeking among more distressed students. In the fully adjusted model reported in Table 5, the distress–attitude association was itself only marginal (*p* = 0.067), so it should be regarded as weak and unstable rather than firmly established.

### 4.4. The Medical-Student Paradox

Medical students report clinically similar levels of self-reported distress to their peers on campus (M = 2.04) and, on average, similar attitude scores (M = 24.70). An earlier draft of this paper proposed that a “medical-student paradox”—a professional-subculture stigma specifically suppressing medical students’ help-seeking—explained these patterns. This is not supported by the present data: the main group comparison was non-significant, and exploratory interaction terms testing whether the stigma–attitude and distress–attitude associations differ by student group were both non-significant (group × stigma, *p* = 0.168; group × distress, *p* = 0.191). There was no group-specific mediation, interaction, or longitudinal test in this study that could demonstrate a medical-student-specific mechanism. We therefore withdraw this interpretation as a conclusion and note it only as an untested hypothesis for future, adequately powered interaction or longitudinal designs [23].

### 4.5. Generalizability

This study offers a picture of mental health attitudes among students from a single university in the Eastern Province of Saudi Arabia (King Faisal University), but its findings should be read within their specific cultural, institutional, and academic context. Given the single-center, convenience-sampled design, we do not extrapolate these findings to Saudi university students as a whole; whether they generalize to other public universities in the Kingdom is an empirical question for future multi-site research, not a claim we make here.

### 4.6. Methodological Observations

The ATSPPH comprises 10 items, of which items 2, 4, 8, 9, and 10 are negatively worded—so a high score on these items signals reluctance to seek professional help, while a high score on the remaining items signals a more favourable attitude toward seeking help. In the pilot, the ATSPPH yielded a Cronbach’s alpha of 0.580 with the negative items reverse-scored in Jamovi, but a higher value (0.687) when they were left unreversed. This likely reflects acquiescence bias: some students, moving quickly through a self-administered questionnaire, agree even with structurally opposing statements. We nonetheless kept the reverse-scored structure to preserve validity. When this short form is used in different cultural contexts, lower internal consistency is frequently reported, hinting at a subtle gap between conceptual help-seeking attitudes and perceived structural barriers.

In the main study, the ATSPPH produced a Cronbach’s alpha of 0.671. As detailed in Section 2.6, this reported negative correlation reflected item 8 being entered in its raw, unreversed form in that specific calculation; correctly reverse-scored (as used throughout this paper’s totals), item 8’s item-rest correlation is weakly positive (r = 0.08), similar to item 4 (r = 0.13) and well below the other items (r = 0.25–0.52). We no longer interpret this item as evidence that time and cost are perceived independently of general attitudes; that claim is not supported once the scoring is corrected. This accords with earlier work: Zhao et al. found that 41% of students reported lack of time, one of the most common barriers to seeking professional help [22], and Alsalman et al. ranked time as the fourth most common barrier—difficulty taking time off from education—with 77.9% considering it a barrier to some degree and 29.9% viewing it as a major one [5]. In El-Hachem et al., positive attitudes and awareness of free on-campus services predicted stronger intentions to seek counseling, while self-stigma predicted weaker attitudes [24]. Time and cost as specific barriers were not directly modeled in this study (item 8 contributes to a total attitude score, not a separate barrier score), so we do not draw a specific conclusion about them here; we note the broader literature above only as context. The item was retained in the primary analysis to preserve the original scale; a sensitivity analysis excluding it is reported in Section 3.4 and did not change the substantive conclusions.

Items 3 and 15 of the HSCL-25 also correlated negatively in the pilot, though not in the original validation study, and we kept both despite this, for the following reasons:

Items H3 (“Faintness, dizziness, or weakness”) and H15 (“Poor appetite”). These two items showed negative item-total correlations in the small pilot sample only. Re-examined in the full sample (n = 580), both items showed positive item-rest correlations (r = 0.49 and 0.38, respectively), consistent with no negative correlations recurring outside the pilot. We therefore no longer offer extended post hoc explanations for the pilot-only anomaly; possible contributors include the small pilot sample size and item-wording sensitivity, but neither was formally tested and both items were retained without modification, as originally planned.

The minor item-total anomalies seen in the pilot resolved once the analysis was scaled up to the full sample. This study draws on a large sample of 580 students, well above the minimum statistical requirement, and its use of validated Arabic versions of the instruments reduced linguistic barriers and let students express themselves accurately. Several limitations should nonetheless be acknowledged. First, because the design is cross-sectional, we cannot establish a causal pathway linking distress, stigma, and long-term help-seeking behavior. Second, recruitment relied on convenience sampling through WhatsApp, which introduces selection bias—evident in the heavy concentration of first-year students in the non-health-related group and a general skew toward female respondents. This combination of a non-probability sample and recruitment through a messaging application warrants closer scrutiny, as it constrains the inferences that can legitimately be drawn from the data in several distinct ways. Because the survey link was forwarded within college WhatsApp groups rather than sent to an enumerated sampling frame, the study has no denominator: the number of students who received or opened the invitation is unknown, no response rate can be computed, and the characteristics of non-responders cannot be compared with those of participants. It is therefore not possible to quantify how far the achieved sample departs from the student population, and the observed proportions should be read as descriptive of this sample rather than as population prevalence estimates. Recruitment through group chats also propagates along existing social networks, so participants are unlikely to be independent of one another in the way that conventional standard errors assume; students within the same college, cohort, or friendship group had correlated probabilities of being invited, which may render the reported confidence intervals somewhat narrower than the true uncertainty. A further concern is self-selection on the study topic itself: an invitation to complete a survey about psychological help-seeking may preferentially attract students who are more interested in, or more comfortable with, mental health issues, and may deter those holding the most stigmatising views—precisely the group of greatest interest. If so, the observed attitudes may be more favourable, and perceived stigma lower, than in the wider student body, and any true between-group difference may have been attenuated. Access is a related issue: although WhatsApp use is near-universal among Saudi university students, distribution was mediated by student representatives and depended on active membership of official college groups, so students who are less socially engaged, on clinical placement, or otherwise peripheral to those networks were systematically less likely to be reached. Finally, because participation was not restricted to verified institutional accounts, eligibility rested on self-report; the 8 respondents (1.4%) who indicated they were not King Faisal University students were retained in the primary analysis and excluded in a sensitivity analysis that did not alter the conclusions, but the possibility that the link was forwarded beyond the intended population cannot be fully excluded. Taken together, these features mean the findings are best interpreted as internally valid associations within a large single-institution sample rather than as estimates generalisable to Saudi university students at large, and replication using probability-based sampling from an institutional enrolment frame would be needed to establish population-level estimates. Third, because the data are self-reported and touch on sensitive topics such as psychiatric history and social stigma, they are subject to social desirability bias. We sought to limit these biases by ensuring anonymity and voluntary participation. Our findings suggest that relying on mental health awareness campaigns alone is unlikely to be effective: students in health-related programmes, who might be expected to be well versed in mental health, did not report significantly more favourable attitudes toward seeking professional psychological help than their non-health-related peers. Institutional effort should instead focus on reducing stigma and logistical barriers. Evans-Lacko et al. argue that the meta-analytic recommendation to target personal attitudes over public opinion is an over-extrapolation that overlooks how community-level stigma feeds internalized personal stigma [25]. We therefore recommend that KFU establish a digital, confidential, and fully anonymous counseling pathway that bypasses perceived stigma (SSRPH). University clinics offering flexible, non-daytime counseling sessions may also help reduce scheduling-related barriers reported in the broader literature [5,22], though this study did not directly measure time or cost as separate barriers and this recommendation is offered as an implication of that literature rather than a finding of the present study. Mental health support should not be a last resort for the severely distressed, but a readily accessible option for every student.

## 5. Conclusions

Among respondents from King Faisal University, no statistically significant difference in attitudes toward seeking professional psychological help was detected between the predefined health-related and non-health-related student groups, in both unadjusted and adjusted analyses. Higher perceived stigma was associated with less favorable attitudes, while psychological distress showed a smaller, positive association that was attenuated once prior use of mental health services was accounted for. These findings should be interpreted cautiously because of the single-center, cross-sectional convenience sample; the imbalance in gender and academic-year composition between groups; the modest internal consistency of the ATSPPH-SF; and the modest variance explained by the regression model (7.9%). With these caveats, the findings are consistent with a continued need for stigma-reduction efforts and confidential, accessible mental health services at King Faisal University, though the specific institutional recommendations offered here should be confirmed by future interventional or longitudinal research before wide implementation.

## Figures and Tables

**Table 1:** Sociodemographic characteristics of participants, by student group (N = 580).

Characteristic	Category	Health-Related (n = 279)	Non-Health-Related (n = 301)	*p*-Value
Gender	Female	152 (54.5%)	230 (76.4%)	<0.001
	Male	127 (45.5%)	71 (23.6%)	
Age (years)	18	20 (7.2%)	73 (24.3%)	<0.001
	19	29 (10.4%)	84 (27.9%)	
	20	54 (19.4%)	60 (19.9%)	
	21	88 (31.5%)	38 (12.6%)	
	22	48 (17.2%)	24 (8.0%)	
	23	22 (7.9%)	7 (2.3%)	
	24	7 (2.5%)	6 (2.0%)	
	25 or older	11 (3.9%)	9 (3.0%)	
Academic year	Preparation	18 (6.5%)	58 (19.3%)	<0.001
	Year 1	43 (15.4%)	113 (37.5%)	
	Year 2	42 (15.1%)	54 (17.9%)	
	Year 3	105 (37.6%)	33 (11.0%)	
	Year 4	38 (13.6%)	32 (10.6%)	
	Year 5	23 (8.2%)	9 (3.0%)	
	Internship	10 (3.6%)	2 (0.7%)	
Nationality	Saudi Arabia	268 (96.1%)	294 (97.7%)	0.378
	Other	11 (3.9%)	7 (2.3%)	
Living area	City	212 (76.0%)	224 (74.4%)	0.734
	Village	67 (24.0%)	77 (25.6%)	
Employment	Employed	10 (3.6%)	15 (5.0%)	0.532
	Not employed	269 (96.4%)	286 (95.0%)	
Marital status	Single, male	125 (44.8%)	70 (23.3%)	<0.001
	Single, female	130 (46.6%)	208 (69.1%)	
	Married, male	1 (0.4%)	1 (0.3%)	
	Married, female	21 (7.5%)	20 (6.6%)	
	Divorced, male	1 (0.4%)	0 (0.0%)	
	Divorced, female	1 (0.4%)	2 (0.7%)	
Economic status	High income	20 (7.2%)	27 (9.0%)	0.693
	Middle income	223 (79.9%)	236 (78.4%)	
	Low income	28 (10.0%)	26 (8.6%)	
	Other	8 (2.9%)	12 (4.0%)	

Data are n (%). Percentages are calculated within each student group (i.e., column percentages, denominators n = 279 for the health-related group and n = 301 for the non-health-related group) so that the two groups can be compared directly; percentages within each characteristic therefore sum to 100% down each group column. The health-related group comprises the Colleges of Medicine, Veterinary Medicine, Applied Medical Sciences, Dentistry, and Clinical Pharmacy; the non-health-related group comprises all remaining colleges. *p*-values are from Pearson chi-square tests comparing the distribution of each characteristic between groups. For marital status, 3 of 12 cells had an expected count below 5, so this *p*-value should be interpreted with caution. This table replaces the three separate sociodemographic tables presented in the previous version of the manuscript.

**Table 2:** Descriptive statistics for the study scales, by group.

Scale	Group	N	Mean	Median	SD	SE
Panel A. Broad track (health-related vs. non-health-related)						
HSCL-25 (mean)	Health-related	279	2.08	2.00	0.573	0.0343
	Non-health-related	301	2.06	2.00	0.607	0.0350
SSRPH (total)	Health-related	279	10.85	11.00	3.216	0.1925
	Non-health-related	301	10.16	10.00	3.341	0.1926
ATSPPH-SF (total)	Health-related	279	24.72	25.00	5.157	0.3087
	Non-health-related	301	25.34	25.00	4.801	0.2767
Panel B. College of Medicine vs. all other colleges						
HSCL-25 (mean)	College of Medicine	147	2.04	1.96	0.559	0.0461
	Other colleges	433	2.08	2.00	0.601	0.0289
SSRPH (total)	College of Medicine	147	11.31	11.00	3.291	0.2714
	Other colleges	433	10.21	10.00	3.257	0.1565
ATSPPH-SF (total)	College of Medicine	147	24.70	25.00	5.266	0.4343
	Other colleges	433	25.15	25.00	4.881	0.2345

**Table 3:** Independent-samples *t*-tests (health-related vs. non-health-related).

Scale	Test	Statistic	df	*p*
HSCL-25 (mean)	Student’s *t*	0.444	578	0.657
	Welch’s *t*	0.445	578	0.657
SSRPH (total)	Student’s *t*	2.555	578	0.011
	Welch’s *t*	2.559	577	0.011
ATSPPH-SF (total)	Student’s *t*	−1.488 ^a^	578	0.137
	Welch’s *t*	−1.484	566	0.138

Note. H_a_: μ(health-related) ≠ μ(non-health-related). ^a^ Levene’s test was significant (*p* < 0.05), indicating a violation of the assumption of equal variances.

**Table 4:** Multiple linear regression predicting ATSPPH-SF total.

Predictor	Estimate	SE	95% CI Lower	95% CI Upper	*t*	*p*	β
Intercept	27.704	0.8848	25.966	29.442	31.31	<0.001	—
Stigma (SSRPH total)	−0.427	0.0615	−0.548	−0.306	−6.94	<0.001	−0.283
Distress (HSCL-25 mean)	0.876	0.3433	0.202	1.551	2.55	0.011	0.104

Outcome: ATSPPH-SF total. N = 580; R = 0.282, R^2^ = 0.079, adjusted R^2^ = 0.076.

**Table 5:** Adjusted multivariable linear regression predicting ATSPPH-SF total (N = 580).

Predictor	B	SE	95% CI	*t*	*p*
Intercept	22.343	3.282	[15.90, 28.79]	6.81	<0.001
Student group (health-related vs. non-health-related)	−0.140	0.438	[−1.00, 0.72]	−0.32	0.749
Gender (male vs. female)	−1.526	0.471	[−2.45, −0.60]	−3.24	0.001
Age (years)	0.271	0.173	[−0.07, 0.61]	1.57	0.117
Academic year 1 (vs. preparation year)	0.715	0.682	[−0.62, 2.05]	1.05	0.295
Academic year 2 (vs. preparation year)	0.587	0.786	[−0.96, 2.13]	0.75	0.455
Academic year 3 (vs. preparation year)	−0.081	0.849	[−1.75, 1.59]	−0.09	0.924
Academic year 4 (vs. preparation year)	1.630	0.971	[−0.28, 3.54]	1.68	0.094
Academic year 5 (vs. preparation year)	−1.279	1.253	[−3.74, 1.18]	−1.02	0.308
Internship year (vs. preparation year)	1.095	1.755	[−2.35, 4.54]	0.62	0.533
High income (vs. middle income)	0.207	0.732	[−1.23, 1.64]	0.28	0.777
Low income (vs. middle income)	−0.620	0.702	[−2.00, 0.76]	−0.88	0.378
Other income (vs. middle income)	−0.579	1.085	[−2.71, 1.55]	−0.53	0.594
Nationality (non-Saudi vs. Saudi)	−1.024	1.156	[−3.29, 1.25]	−0.89	0.376
Living area (village vs. city)	−0.202	0.460	[−1.11, 0.70]	−0.44	0.660
Perceived stigma (SSRPH total)	−0.370	0.063	[−0.49, −0.25]	−5.87	<0.001
Psychological distress (HSCL-25 mean)	0.657	0.358	[−0.05, 1.36]	1.83	0.067

Outcome: ATSPPH-SF total score. Higher scores indicate more favourable attitudes toward seeking professional psychological help. B = unstandardised regression coefficient; CI = confidence interval. Reference categories: non-health-related group, female, preparation year, middle income, Saudi nationality, city residence. N = 580; R^2^ = 0.128, adjusted R^2^ = 0.103; F(16, 563) = 5.17, *p* < 0.001. All 580 participants had complete data on every model variable. Variance inflation factors ranged from 1.02 to 3.41, indicating no problematic multicollinearity.

## Data Availability

All data are available upon request from the corresponding author. The data are not publicly available due to privacy restrictions.

## Supplementary Materials

The following supporting information can be downloaded at: https://www.mdpi.com/article/10.3390/healthcare14172704/s1, Table S1. Exploratory factor analysis (principal-axis factoring, varimax rotation) and item-rest correlations for the 10-item Attitudes Toward Seeking Professional Psychological Help–Short Form (ATSPPH-SF), N = 580.
