# Supplementary material for: Attitudes Toward Seeking Professional Psychological Help Among Health-Related and Non-Health-Related Students: A Cross-Sectional Study
Source: Healthcare (Basel). 2026 Aug 25;14(17):2704. doi: 10.3390/healthcare14172704 (PMC13565295; doi:10.3390/healthcare14172704)
Supplement: Supplementary file 1 [file healthcare-14-02704-s001.zip › healthcare-4433163-supplementary.pdf]

Supplementary Table S1

Exploratory factor analysis (principal-axis factoring, varimax rotation) and item-rest correlations for the 10-item Attitudes Toward Seeking Professional Psychological Help – Short Form (ATSPPH-SF), N = 580.

Item	ATSPPH-SF item (abbreviated)	Factor 1	Factor 2	Factor 3	h^2	Item-rest r
1	If I believed I was having a nervous breakdown, my first inclination would be to get professional attention.	0.603	−0.012	0.138	0.383	0.392
2	The idea of talking about problems with a psychologist strikes me as a poor way to get rid of emotional conflicts. (R)	0.175	0.386	0.292	0.265	0.354
3	If I were experiencing a serious emotional crisis at this point in my life, I would be confident that I could find relief in psychotherapy.	0.661	−0.062	0.186	0.475	0.393
4	There is something admirable in the attitude of a person who is willing to cope with his or her conflicts and fears without resorting to professional help. (R)	−0.063	0.445	−0.137	0.221	0.129
5	I would want to get psychological help if I were worried or upset for a long period of time.	0.728	0.147	−0.017	0.552	0.517
6	I might want to have psychological counseling in the future.	0.653	0.103	−0.122	0.452	0.428
7	A person with an emotional problem is not likely to solve it alone; he or she is likely to solve it with professional help.	0.619	0.107	−0.278	0.473	0.383
8	Considering the time and expense involved in psychotherapy, it would have doubtful value for a person like me. (R)	−0.098	0.259	0.484	0.311	0.080
9	A person should work out his or her own problems; getting psychological counseling would be a last resort. (R)	0.142	0.741	0.052	0.572	0.423
10	Personal and emotional troubles, like many things, tend to work out by themselves. (R)	0.006	0.506	0.171	0.286	0.247
	**Sum of squared loadings**	**2.204**	**1.267**	**0.517**		
	**% of total variance**	**22.0**	**12.7**	**5.2**		

Note. All items were scored so that higher values indicate more favourable attitudes toward seeking professional psychological help; items marked (R) were reverse-scored before analysis. Item-rest r = correlation between the item and the sum of the remaining nine items. h^2 = communality. Loadings ≥ 0.40 in absolute value are conventionally regarded as salient. Kaiser–Meyer–Olkin measure of sampling adequacy = 0.76; Bartlett's test of sphericity $\chi^2(45) = 1143.4$, $p < 0.001$. Initial eigenvalues were 2.83, 1.88, and 1.09 for the three retained factors (28.3%, 18.8%, and 10.9% of total variance; 58.0% cumulative). Parallel analysis against 500 random datasets of equivalent dimensions retained two factors rather than three. Cronbach's α for the full 10-item scale = 0.671; α = 0.696 with item 8 removed.

Items 4 and 8 show the weakest association with the remainder of the scale (item-rest r = 0.129 and 0.080 respectively) and the lowest communalities, and item 8 loads primarily on a small third factor. Factor 1 is defined by the positively worded help-seeking items (1, 3, 5, 6, 7) and Factor 2 by the negatively worded self-reliance items (2, 4, 9, 10), indicating that the multi-factor structure largely reproduces the direction of item wording rather than distinct substantive dimensions. These results are the basis for the caution expressed in the main text regarding the treatment of the ATSPPH-SF as a single unidimensional total score.
